# Service use and costs for people with headache: a UK primary care study

**DOI:** 10.1007/s10194-011-0362-0

**Published:** 2011-07-09

**Authors:** Paul McCrone, Paul T. Seed, Andrew J. Dowson, Lucy V. Clark, Laura H. Goldstein, Myfanwy Morgan, Leone Ridsdale

**Affiliations:** 1 P024, Health Service and Population Research Department, Institute of Psychiatry, King’s College London, De Crespigny Park, London, SE5 8AF UK; 2 Division of Reproduction and Endocrinology, King’s College London, London, UK; 3 King’s College Hospital, London, UK; 4 Wolfson Institute of Preventive Medicine, Queen Mary University of London, London, UK; 5 Department of Psychology, Institute of Psychiatry, King’s College London, London, UK; 6 Department of Public Health Sciences, King’s College London, London, UK; 7 Department of Clinical Neuroscience, Institute of Psychiatry, King’s College London, London, UK

**Keywords:** Economics, Costs analysis, Primary care, Headache

## Abstract

This paper aims to estimate the service and social costs of headache presenting in primary care and to identify predictors of headache costs. Patients were recruited from GP practices in England and service use and lost employment recorded. Predictors of cost were identified using regression models. Service and social costs were available on 288 and 282 patients, respectively. Average service costs over 3 months were £117 whilst total costs (including lost production) were £582. Patients referred to neurologists had service costs that were £82 higher than those not referred (90% CI £36–£128). Costs including lost employment were higher by £150, but this was not significant (90% CI -£139–£439). The annual mean service and social costs, weighted to represent population rates of referral, were £468 and £2328, respectively. Higher costs were significantly related to pain. Age was linked to higher service costs and lower social costs. The figures extrapolated to the whole of the UK suggest £956 million due to service use and £4.8 billion including lost employment. These are likely to be underestimates because many people experiencing headaches do not consult their GP.

## Introduction

Headache, including migraine, is a common problem and is in the top ten causes of disability [1]. It is usually self-managed, but had been estimated to account for 44 consultations per 1,000 people in primary care. Similarly, although GPs refer to neurologists only 2–3% of patients consulting for headaches, this condition accounts for up to one-third of new specialist neurology appointments in the UK [2].

Clearly headache can cause distress for individuals and limit their activities. This, combined with the demand for treatment, suggests that there is an economic burden associated with headache [3]. Direct costs are due to the use of services, such as doctor time and medication in order to treat the headache. Indirect costs are caused by the impact that headache has on the activities of the patient, and are typically confined to the effect on productivity. A number of studies have sought to estimate headache (mainly migraine) costs, but these show considerable variations in methods and findings [4]. In Europe, the costs of migraine and other headaches have been estimated to be €590 per person, of which 94% was due to indirect costs [5]. In the United States, the total cost of migraine was estimated at $14.4 billion, again with most (93%) due to indirect costs [6]. A recent review of studies from the USA has also suggested that the indirect costs of migraine outweigh the direct costs, although with the difference slightly less compared to the above two studies [7]. In another study, the costs of treating migraine in Brazil have been estimated at $140 million, with one-third of this due to primary care services [8].

Most studies have reported costs for migraine, but this is only a subset of all headaches treated in primary care settings. We have previously compared the demographic and clinical characteristics of patients referred by GPs to neurologists with those remaining in primary care. It was found that referral to neurologists was not related to headache severity, but was associated with an increased number of consultations with GPs and increased fear and anxiety expressed by patients regarding their headaches, as well as with GPs lack of clinical confidence and patient pressure [9, 10]. The aims of this paper are to: (1) measure the service use and costs for people with headache presenting in primary care, (2) compare service use and costs for patients referred and not referred to specialists, and (3) identify patient characteristics associated with costs.

## Method

This was a primary care-based study set in England and the study methodology has been described in detail elsewhere [9]. Patients were recruited from 18 general practices in the south Thames region––a region covering urban and rural areas. The number of patients aged 18–75 registered with GPs was around 141,100. GP practices were recruited over a 1-year period. As GPs only refer a small proportion of patients to specialists, it was decided to over-sample these. Consequently, any patient referred to specialist care during a 1-year period was eligible for the ‘referred’ group, whilst patients presenting with headache during a 7-week period and who remained under the care of the GP (a far more common scenario) were identified by someone in each practice and were eligible for inclusion in the ‘consulted but non-referred group’. For inclusion, headache was classified according to diagnostic codes used by UK primary care practices (i.e. Read Codes). Patients were potentially included if the diagnostic codes referred to ‘headache’ or ‘migraine’. Patients were excluded if there were secondary causes of the headache, if the patient was unable to participate in the interviews due to cognitive impairment, or if they were unable to read and/or write English. Informed written consent was obtained from participants.

Eligible patients were invited to be interviewed by research workers in their homes or at the general practice. The interviews consisted of a number of measures including the migraine disability assessment score (MIDAS) [11], the Headache Impact test (HIT)-6 [6, 12] the Hospital Anxiety and Depression Scale (HADS) [13], and the Revised Illness Perception Questionnaire (IPQ-R) [14]. These measures are described in detail by Ridsdale et al. [9]. Seven subscales from the IPQ-R were used in the analyses: timeline acute-chronic, timeline cyclic, consequences (where high scores represent strong beliefs about the chronicity, duration and impact of headache), personal control, treatment control, illness coherence, and emotion (where high scores represent strong beliefs about how well the individual believes that they or formal treatments can control headaches and how well the person understands and is troubled emotionally by their headache disorder).

### Service use and costs

It is important to take a comprehensive approach when estimating the economic consequences of a particular condition [15]. Headache is no exception, as patients will potentially be accessing a range of different services and may also take time off work. A societal perspective was adopted in this study. We collected information on whether specific services had been used in the previous 3 months, and if so how many times. The services included were: contacts with general practitioners (GPs), neurologists, other medical specialists, contacts with other professionals (including complementary healthcare), scans undertaken (MRIs and CTs), and prescribed medication. For scans and GP contacts we asked about the number of times these had been received for headache and how many for other reasons. Unit costs were attached to the information on service use using nationally applicable figures [16, 17].

### Productivity costs

Headache can have an impact through people taking time off work, or having reduced work effectiveness. The MIDAS questionnaire includes two questions covering the effect of lost work time due to headache. The first of these asks for the number of whole days lost from work in the past 3 months. The second asks for the number of days where productivity is reduced by more than 50%. In the absence of further information, we have conservatively assumed this to be equal to half-a-day’s lost work. The economic cost of lost work time was calculated by multiplying the lost days by the earnings that patients in the sample received (calculated as a daily figure). Not all patients stated their earnings and in these cases we obtained average figures for their job type and gender from official data [18]. Where the patients stated that they were working, but did not provide information on their earnings or job type, we used a gender-specific average based on the other sample members. There is some controversy about the best approach to take in calculating the productivity costs. In times of high unemployment, it may be that prolonged absence from work does not result in a cost because someone else can be taken on. However, with headache it is more likely that lost work time will be sporadic and short-term and replacement by someone else would be rare. A further area of contention is the use of the wage rate as the value of lost work. This assumes that wages reflect true economic value, but due to market imperfections this may of course not be true. Given these issues, the resulting productivity costs must be treated with caution.

### Analysis

Service use and costs were reported for each group. The overall costs are not representative of the population of people consulting GPs due to the overrepresentation of those referred to specialists. Therefore, weighted costs were also computed, based on the finding by Latinovic et al. [2] that over a year 2% of consultations result in a referral to a neurologist. The significance of the difference in total costs with and without productivity costs was assessed using regression models. Such models allow the impact of independent variables (in this case being referred or not) on a dependent variable (cost) to be determined. We constructed 90% confidence intervals partly because the analysis was exploratory and also because we recognised that different levels of risk may be acceptable when assessing cost differences compared with comparing clinical differences.

Further regression models were then constructed to identify the demographic and clinical characteristics that were associated with differences in costs in addition to any difference due to whether the patient was referred or not. Independent variables in the model related to headache severity (number of headache-related symptoms, severity of pain, need to lie down when headaches occur, feeling fed up due to pain), psychological distress (anxiety and depression scores from HADS) [19], illness perception subscales, age, gender, and the referred/not referred variable. The total score from the HIT-6 was not used as some items directly relate to lost work time, which has already been included in the costs. Some variables had missing values and, therefore, we used imputation methods using the other variables (plus whether the patients considered the headaches to be psychological in cause) in Stata v10 to estimate these values. Because we oversampled patients who were referred we adjusted the confidence intervals around the regression coefficients by again weighting the observations. If we again assume that 98% of patients do not get referred then the weight for each non-referred patient is 0.384 (i.e. 98 divided by 255 patients) and the weight for each referred patient is 0.042 (i.e. 2 divided by 48 patients).

## Results

During the sampling periods there were 533 patients who consulted a GP for headache and a further 93 who were referred to secondary care services. Of these 626 patients, 569 met inclusion criteria and 303 consented to take part (255 non-referred, 48 referred). These patients were predominantly female (71% of non-referred patients, 64% referred patients) and of similar mean age (39 vs. 41). Ridsdale et al. [9] show that the two groups do not differ significantly in terms of MIDAS, HIT-6, or HADS scores.

Most patients had consulted their GP during the previous 3 months, but for the majority this was not due to headache (Table 1). The average costs of GP contacts for all reasons and specifically for headache were higher for those who had been referred to specialists. A small number of patients in the non-referred group did have neurologist contacts during the previous 3 months, possibly for other conditions, whereas one quarter of the referred patients had seen neurologists. Most patients in both groups were taking some form of medication that was likely to be headache related. MRI or CT scans were more likely to have been received by referred patients.

**Table 1:** Use and cost of services and lost employment during previous 3 months

	Non-referred patients			Referred patients			All patients			
	*N* (%) patients	Mean (SD) contacts/days	Mean (SD) cost	*N* (%) patients	Mean (SD) contacts/days	Mean (SD) cost	*N* (%) patients	Mean (SD) contacts/days	Mean (SD) cost	Weighted mean cost
GP contacts for all reasons	209 (82)	1.9 (1.6)	36 (31)	37 (79)	2.5 (3.0)	48 (57)	246 (82)	2.0 (1.9)	38 (37)	36
GP contacts for headache	81 (32)	0.5 (0.9)	9 (16)	21 (45)	0.9 (1.5)	17 (29)	102 (34)	0.5 (1.0)	10 (19)	9
Neurologist contacts	10 (4)	0.05 (0.3)	8 (41)	12 (25)	0.3 (0.6)	43 (86)	22 (7)	0.1 (0.4)	13 (52)	9
Other professionals	40 (16)	0.7 (2.2)	21 (75)	9 (19)	0.4 (1.0)	20 (55)	49 (16)	0.6 (2.0)	21 (72)	21
Medication	190 (75)	–	31 (89)	40 (83)	–	24 (56)	230 (76)	–	30 (84)	31
MRI for all reasons	13 (5)	0.05 (0.22)	16 (70)	7 (16)	0.16 (0.37)	48 (114)	20 (7)	0.07 (0.25)	21 (79)	17
MRI for headache	11 (4)	0.04 (0.21)	14 (64)	7 (16)	0.16 (0.37)	48 (114)	18 (6)	0.06 (0.24)	19 (75)	15
CT for all reasons	6 (2)	0.02 (0.15)	2 (16)	10 (22)	0.22 (0.42)	23 (43)	16 (6)	0.06 (0.23)	6 (23)	2
CT for headache	4 (2)	0.02 (0.13)	2 (13)	8 (18)	0.18 (0.39)	18 (39)	12 (4)	0.04 (0.20)	4 (20)	2
Lost employment (full days)	88 (38)	2.2 (6.7)	269 (1030)	15 (36)	2.8 (8.6)	211 (604)	103 (36)	2.3 (7.0)	261 (978)	268
Lost employment (part days)	90 (37)	3.5 (9.4)	200 (679)	20 (48)	5.1 (7.5)	312 (523)	110 (39)	3.8 (9.1)	217 (659)	202
Total service cost	237 (97)	–	115 (163)	43 (98)	–	197 (174)	280 (97)	–	128 (167)	117
Total cost	240 (99.6)	–	579 (1405)	41 (100)	–	729 (967)	281 (99.6)	–	601 (1349)	582

Costs in 2003/4 £s

The mean service and total costs for the whole sample are £128 and £601, respectively. For the non-referred patients the figures are £115 and £579, respectively, whilst for those referred they are £197 and £729, respectively. If we weight the costs for non-referred and referred patients by 0.98 and 0.02, respectively, the mean service cost becomes £117 whilst the mean total cost is £582. If representative, this would suggest annual service costs of £468 and total costs of £2,328 per person.

Mean service costs (i.e. excluding lost productivity) for the 3-month period were £82 higher in the referred group, a difference that was statistically significant (90% CI £36–£128). Those who were referred also had more time off work due to headache and this increased the cost difference to £150. However, there was substantial variation in lost work time and this cost difference was not statistically significant (90% CI -£139–£439). Productivity costs accounted for 81% of the costs in the non-referred group and 72% of the costs in the referred group.

**Fig. 1:**
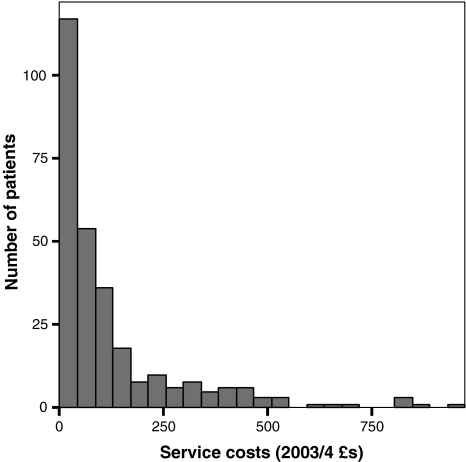
Distribution of service costs

**Fig. 2:**
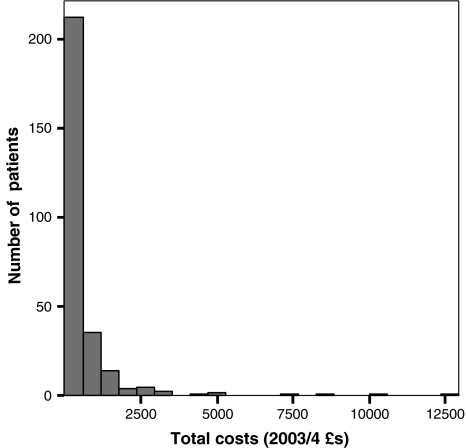
Distribution of total costs

Figures 1 and 2 show the variation in the service costs and total costs. Ten percent of patients accounted for 42 and 57% of service costs and total (including lost production) costs, respectively. The first regression model showed that after controlling for demographic and clinical characteristics service costs were on average £55 higher for patients (i.e. lower than the unadjusted figure of £82) who were referred to specialists, and this was statistically significant (Table 2). Higher service costs were also associated with being male (£44 higher costs than for women), age (each year accounting for an increase in costs of £2), the level of pain they experienced, and how substantially the patient believed the consequences of their headache disorder to be. This model could explain 17% of cost variation. Costs including lost work time were significantly lower for older patients (on average by £10 for each extra year of age), higher for patients with more anxiety (a one unit increase associated with costs that were £52 higher), and higher for patients with stronger beliefs that their headache can be controlled by treatment. Costs were again associated with pain. The model could explain 7% of variation in cost.

**Table 2:** Weighted regression of analysis of service costs and total costs

	Service costs		Total costs	
	Coefficient	90% CI	Coefficient	90% CI
Referred	55	2 to 108	121	−177 to 418
Male	44	1 to 88	−70	−376 to 235
Age	2	1 to 4	−10	−19 to −2
Anxiety score	6	0 to 11	52	11 to 94
Depression score	2	−5 to 10	−12	−66 to 41
Severity of pain (HIT 1)	12	2 to 23	96	6 to 185
Need to lie down (HIT 3)	−6	−18 to 6	−44	−117 to 30
Feel fed up due to pain (HIT 5)	1	−7 to 10	24	−53 to 102
IPQ-R number of headache-related symptoms	1	−2 to 5	−1	−27 to 25
IPQ-R timeline acute-chronic	−2	−6 to 3	11	−29 to 50
IPQ-R timeline cyclic	0	−5 to 5	−39	−87 to 9
IPQ-R consequences	5	1 to 10	22	−11 to 55
IPQ-R personal control	−3	−8 to 2	12	−31 to 55
IPQ-R treatment control	3	−3 to 10	55	4 to 106
IPQ-R illness coherence	−3	−7 to 0	−3	−32 to 26
IPQ-R emotional representation	1	−4 to 6	−7	−45 to 31
Constant	−218		−1285	
	*R* ^2^ = 0.1668		*R* ^2^ = 0.0729	

Costs in 2003/4 £s

Men had lower total costs than women, although the difference was not significant. This result was surprising as the data revealed that men had slightly more days when they could not work (according to the MIDAS questions) than women (4.5 vs. 4.0 days). Also daily wages were slightly higher for men (£127 vs. £103). Further analyses did though explain the lower total costs for men. For women in employment, there was a positive correlation (0.15) between their daily wages and days off work. However, for men the correlation was negative and larger in magnitude (−0.32). This implies that men who had days off work were likely to be paid less and, therefore, this reduces the overall cost of lost employment, and consequently total costs, compared to women.

## Discussion

Headache is a common health problem affecting over 90% of the population during their lives. Most people self-manage symptoms with over-the-counter medication, with 4% of adults consulting their GP for headache each year [2]. If we extrapolate the 3-month costs then the annual services costs are £468 and the annual total costs are £2,328. When we consider the number of people presenting with headache the costs are substantial. If we apply the above figure of 4% of people consulting their GP with headache to the UK population aged 15 and over (which in 2009 was estimated at 51.1 million [20]), then the service costs for people consulting with headache are £956 million and the total costs including lost production are £4.8 billion.

The results presented in this paper are of importance to a specialist audience because, whilst most patients with headache receive care from GPs, patients with headache account for around one-third of referrals to neurologists [2]. In addition, GPs are increasingly developing special interests including in headache and, therefore, the distinction between ‘specialists’ and ‘generalists’ may be coming less clear [21].

Clearly, the main economic effect of headache is the impact that it has on productivity. We found that 81% of the total costs of headache were due to lost work time. This may be an underestimate of the broader social costs––there would clearly be an impact on work in the home also, as well as on leisure activities. Other studies have found higher proportions accounted for by indirect costs [5, 6], and the difference may be due to the primary care focus of this study and the fact that we included all headache types. In Denmark, headache has been shown to account for 20% of sickness absence [22].

Service costs were found to be higher for patients who had been referred to secondary care services. These extra costs consisted of more GP contacts, contacts with neurologists and scans. The study found that one-quarter of the referred sample had received input from neurologists by the time the first questionnaire was administered. Therefore, the cost difference would be even greater in the longer term once all referred patients had received specialist advice or care.

The regression model found that higher service costs were significantly associated with age, which likely reflects increasing morbidity with age. Greater pain severity was also associated with higher costs. The only IPQ-R subscale that was significantly related to service cost was beliefs about the consequences of the headache. The more substantial the consequences were perceived to be, the higher the costs. Referrals to neurologists for headache has been associated with dissatisfaction in up to one-third of patients [23], and this is possibly because underlying anxieties have not been addressed. Cognitive-behavioural approaches may be appropriate in these circumstances, and it will be worthwhile to explore and develop approaches which include such techniques [24].

Each of the above relationships could be expected and indicate that patients with higher levels of need are in receipt of most resources. Total costs were, however, inversely related to age, which is due to the fact that older patients are less likely to be in employment and to experience lost work days. As before, pain severity was associated with higher costs.

The weighted mean cost for neurologist contacts was only £9, but across the population this would equate to around £18 million. In a recent study, we have shown that if GPs receive specific training in headache management, then patients can be seen for a lower cost, and they are more satisfied with the service [21]. However, for younger patients referral may be more socially cost-effective provided it does lead to amelioration of symptoms which cause distress and disability. This is worth testing, and might justify increased investment in providing more intermediate or specialist services.

### Limitations

The study has a number of limitations. First, a limited number of services were included in the costings and as such the figures may be an underestimate. Second, we have included GP and neurologist contacts and interventions used by people with headache, but not necessarily due to headache. It is clear that comorbidity is common and does have a cost impact [25], but it is problematic to disentangle this effect from costs unrelated to headache. Third, patients were recruited over a 1-year period and if they were towards the end of this period then they may not yet have seen a neurologist even if referred. Conversely, some patients may have been defined as non-referrals, but may actually have seen a neurologist following a referral before the recruitment period. Fourth, the sample only consists of those who contacted their GP. Substantial numbers of people do not seek treatment and, whilst this means they do not incur service costs, it is most likely that a number take time off work. Fifth, days where the productivity was reduced by at least 50% were costed using half of the daily wage rate. This approach, therefore, possibly underestimates the productivity costs. In addition, we have not included days where the productivity was reduced, but by less than 50%. This seems reasonable and the productivity costs remain substantial.

## Conclusions

This paper has found that the mean costs associated with headache presenting in primary care are £601 over a 3-month period. Most of this is due to lost employment. Service costs are significantly higher for patients referred to neurologists and those with greater symptom severity.
